# Cerebral pulse oximetry in carotid endarterectomy: an impact study

**DOI:** 10.1590/1806-9282.20250771

**Published:** 2025-10-27

**Authors:** Mehmet Ali Yürük, Aşkın Kılıç, Ufuk Sayar, Ali Kemal Arslan, Muhammet Onur Hanedan

**Affiliations:** 1 Karadeniz Technical University, Faculty of Medicine, Department of Cardiovascular Surgery – Trabzon, Turkey.; 2 Samsun Education and Research Hospital, Department of Cardiovascular Surgery – Samsun, Turkey.; 3 Ahi Evren Thoracıc and Cardiovascular Surgery Traınıng and Research Hospıtal, Department of Cardiovascular Surgery – Trabzon, Turkey.

**Keywords:** Oximetry, Carotid endarterectomy, Intraoperative monitoring, Brain ischemia

## Abstract

**OBJECTIVE:**

The aim of this study was to evaluate the effectiveness of cerebral pulse oximetry in improving early results (30 days) of surgical outcomes and patient safety during carotid endarterectomy, focusing on its role in guiding selective shunt use and reducing procedure-related complications.

**METHODS:**

A retrospective review was conducted on 179 patients who underwent carotid endarterectomy at a cardiovascular surgery clinic (2010–2020). The study emphasized adherence to ethical standards, rigorous data analysis, and stringent participant inclusion criteria and evaluated the impact of cerebral pulse oximetry on surgical outcomes.

**RESULTS:**

Cerebral pulse oximetry significantly influenced intraoperative decisions. Mean total operative time and cross-clamp duration were significantly reduced in the group cerebral pulse oximetry (66.96±8.21 vs. 57.41±7.24 min and 28.68±4.20 vs. 18.63±3.91 min, respectively). Routine shunt placement was also lower in this group, with usage reported in only 0.9 vs. 47.2%.

**CONCLUSION:**

Cerebral pulse oximetry during carotid endarterectomy may reduce the necessity for routine shunt placement, minimizing associated risks and enhancing operative efficiency.

## INTRODUCTION

Carotid endarterectomy (CEA) is a vital surgical intervention to reduce the risk of stroke in patients with significant carotid artery stenosis. The success of this procedure hinges on the meticulous management of cerebral perfusion during the operation^1^. Cerebral oximetry has recently emerged as a valuable tool for monitoring cerebral oxygenation, offering the potential to enhance surgical outcomes^2^. It employs noninvasive technology that measures oxygen saturation in brain tissue during carotid artery surgery, providing essential insights into cerebral perfusion and oxygenation status^3^. This technology is especially important in detecting intraoperative cerebral ischemia—one of the most serious complications associated with CEA^4^. Using cerebral oximetry significantly reduces the incidence of postoperative neurological deficits^5^. The intraoperative use of shunts to maintain cerebral blood flow remains a subject of debate among vascular surgeons. While shunting aims to preserve perfusion during arterial clamping, it carries inherent risks. Current studies indicate that cerebral oximetry may guide individualized decision-making regarding shunt placement, thereby minimizing unnecessary shunt use and its associated complications^6^. In this study, we aimed to assay cerebral oximetry on shunt utilization, operative efficiency, and early period consequences of postoperative outcomes after 30 days.

## METHODS

This retrospective cohort study was conducted at Ahi Evren Chest, Cardiovascular Surgery Training and Research Hospital between September 2010 and September 2020. The primary objective was to evaluate the efficacy and clinical outcomes associated with cerebral pulse oximetry (CPO) in patients undergoing CEA. Ethical approval for the study was obtained from the Ethics Committee of Kanuni Training and Research Hospital (Approval Date: 17/12/2020; Approval Number: 2020/83).

The study population comprised adult patients aged 18 years and older diagnosed with carotid artery stenosis who underwent CEA during the specified period. Patients were categorized into two groups based on whether they used the intraoperative monitoring method or not. In our clinic, there was no possibility of monitoring cerebral oxygenation between 2010 and 2014. Since 2015, the cerebral pulse oximeter-CPO (INVOS™ 5100C regional oximeter, Somanetics Corporation, Troy, MI) has been used during surgical procedures. The patients included in this study were divided into two groups: Not using CPO (N-CPO) versus using CPO (CPO). All patients were operated on by the same surgical team.

Medical records were thoroughly reviewed to extract data on patient demographics, clinical characteristics, surgical parameters, and postoperative outcomes, including complications such as bleeding, as well as minor and major postoperative events. Patients with incomplete medical records—especially in relation to critical variables—or those for whom CPO could not be applied due to contraindications (e.g., dermatological conditions interfering with sensor placement, diseases such as allergic skin history, scleroderma, skin structural disorders, and psoriasis) were excluded from the analysis. CPO monitoring was conducted using devices calibrated in accordance with manufacturer specifications. A standardized protocol was followed, involving placing sensors on the forehead to continuously monitor cerebral oxygen saturation throughout the surgical procedure. Data were recorded and analyzed to assess the influence of cerebral oxygenation on intraoperative decision-making and postoperative outcomes.

Operation procedure and desaturation criteria: Two separate sensors were placed on the right and left of the patient's frontal region before medication and baseline values were recorded. The desaturation value that developed after anesthesia medication was accepted as a 20% decrease in the baseline value. In case of desaturation, firstly mean arterial pressure was increased by a minimum of 10 mmHg, and the FiO^2^ value was increased by 10%. Simultaneously, anesthesia was continued with pentothal and mannitol, and decorticate treatment was applied to prevent possible cerebral edema. In cases of resistant desaturation, patients were shunted. In our clinical practice, when CPO was not used, a shunt was applied to all patients with lesions of 10% or more on the contralateral side.

All procedures performed in the study followed institutional and national ethical standards, as well as the principles of the Declaration of Helsinki and its subsequent amendments. Informed consent was obtained from all participants. Informed consent forms are obtained from patients before all medical interventions. This surgical consent form includes approval that medical data can be used in retrospective studies.

All statistical analyses were conducted using the Statistical Package for the Social Sciences (SPSS v26). Normality assumptions were examined with kurtosis and skewness values. If the kurtosis and skewness coefficients were within ±1.5 points, the data were accepted as if normally distributed. Continuous variables were expressed as mean±standard deviation, while categorical variables were presented as frequencies and percentages. The independent samples t-test was employed to compare normally distributed continuous variables between the two groups. Pearson's chi-square test was used to analyze differences in categorical variables. The effects of smoking and hypertension on operative time and cross-clamp time were examined with linear regression analysis. The effects of smoking and hypertension on cerebrovascular accident (CVA) and transient ischemic attack (TIA) were evaluated with binary logistic regression analysis. A p<0.05 was considered statistically significant for all comparisons.

Post-hoc power analysis was performed with G*Power 3.1.9.7 software. Cross-clamp time was used as a reference in calculating the effect size and was 2.49. Accordingly, the study had full power (100%) according to the two-tailed post-hoc results of the study.

## RESULTS

The primary finding of the study was the significant impact of CPO on surgical outcomes in CEA, particularly in Group CPO, where its use was associated with reduced shunt usage and improved operative efficiency. The demographic and clinical characteristics of 179 patients involved in the study were evaluated. The prevalence of hypertension was significantly higher in Group CPO (82.8%) than in Group N-CPO (67.5%) (p=0.017). No significant differences were observed in other baseline demographic parameters. The most common preoperative clinical presentation was CVA, with similar incidence in both groups (45.1 vs. 47.0%; p=0.533). A detailed summary of baseline characteristics is presented in Table 1.

**Table 1:** Demographic and clinical characteristics.

Variables		N-CPO (n=74)	CPO (n=105)	p
Age		70.82±9.32	70.30±8.21	0.694*
Gender				
	Male/female (%)	70.2	80.0	0.133**
	Smoking (%)	48.6	33.3	0.039**
	Hypertension (%)	67.5	82.8	0.017**
	Diabetes mellitus (%)	39.1	31.4	0.283**
	Chronic renal failure (%)	10.8	5.7	0.467**
	Coronary artery disease (%)	62.1	59.0	0.675**
	Peripheral arterial disease (%)	13.5	9.5	0.130**
	Chronic obstructive pulmonary disease (%)	24.3	18.0	0.311**
Symptom (%)				
	Asymptomatic	18.5	20.4	0.534**
	Cerebrovascular event	36.4	32.6	0.519**
	Trans ischemic attack	45.1	47.0	0.533**
Lesion side (%)			
	Right***	58.1	67.6	0.193**
	Left***	60.8	56.1	0.537**

* Independent sample t-test.

** Pearson's chi-square test.

*** Patients with 50% or more lesions in the carotid artery were recorded. N-CPO: non-cerebral pulse oximetry; CPO: cerebral pulse oximetry.

Operative time and cross-clamp (CC) duration were both significantly shorter in Group CPO in 7 days (57.41±7.24 vs. 66.96±8.21, p<0.001, and 18.63±3.91 vs. 28.68±4.20, p<0.001, respectively). A statistically significant reduction in shunt usage was observed in Group CPO (0.9%) compared to Group N-CPO (47.2%) (p<0.001). No significant difference was found between groups regarding the use of patch closure (Table 2).

**Table 2:** Surgical procedure and postoperative outcomes.

Variables		N-CPO (n=74)	CPO (n=105)	P
Operation time (min)		66.96±8.21	57.41±7.24	<0.001*
Cross clamp time (min)		28.68±4.20	18.63±3.91	<0.001*
Shunt (%)		47.2	0.9	<0.001**
Patch (%)		72.9	75.2	0.733**
7-day results				
	Cerebrovascular event (%)	0	0	NEO
	Trans ischemic attack (%)	5.4	0.9	0.161**
	Mortality (%)	0	0.9	1.00**
	Bleeding (%)	8.1	10.4	0.595**
30-day results				
	Trans ischemic attack (%)	2 (2.70)	1 (1.00)	0.570
	Cerebrovascular event (%)	1 (1.40)	1 (1.00)	1.00
	Mortality (%)	1 (1.40)	1 (1.00)	1.00

* Independent sample t-test.

** Pearson's chi-square test. NEO: no events observed; N-CPO: non-cerebral pulse oximetry; CPO: cerebral pulse oximetry.

Regarding postoperative neurological outcomes, no CVA occurred in either group. Although the incidence of TIA was lower in Group CPO (0.9%) compared to Group N-CPO (5.4%), the difference was not statistically significant. One mortality occurred in Group CPO, attributed to a myocardial infarction (MI). The patient with ST elevation and increased troponin level was taken to the angiography laboratory with a preliminary diagnosis of MI. Despite the cardiac intervention procedure, the patient died (Table 2).

When the 30-day results were examined, the incidence of TIA in the N-CPO group was 2.70%. The incidence of both CVA and mortality was 1.40%. In the CPO group, the incidences of TIA (1.00%), CVA (1.00%), and mortality (1.00%) were seen in only one person each. No difference was observed between the groups in terms of the above parameters (p>0.05 for all).

The effects of smoking and hypertension on the operation time and the CC time were investigated with two separate linear regression models in Table 3. It was seen that smoking and hypertension did not significantly affect the operation time and CC time in both models (p>0.05). Also, the effects of smoking and hypertension on the presence of CVA and TIA were investigated with binary logistic regression models. According to the results, the effects of smoking and hypertension on TIA in patients were not found to be statistically significant for both events (p>0.05) (Table 3).

**Table 3:** Regression analysis of the effects of smoking and hypertension on operation time, cross clamp duration, cerebrovascular events, and transient ischemic attack.

		β_0_ (95%CI)	SE	β_1_	p	VIF
Model 1: Operation time						
	Constant	62.118 (59.131–65.105)	1.513		<0.001	
	Smoking	1.686 (-1.017 to 4.39)	1.370	0.092	0.220	1.004
	Hypertension	-1.868 (-4.989 to 1.253)	1.581	-0.088	0.239	1.004
Model 2: Cross clamp time						
	Constant	23.639 (21.542–25.736)	1.063		<0.001	
	Smoking	1.903 (0.005–3.801)	0.962	0.146	0.065	1.004
	Hypertension	-2.106 (-4.297 to 0.085)	1.110	-0.140	0.059	1.004

		Univariate		Multivariate	
		OR (95%CI)	p	OR (95%CI)	p
Model 1: Cerebrovascular event					
	Smoking	1.223 (0.633–2.364)	0.549	1.204 (0.622–2.333)	0.582
	Hypertension	0.74 (0.352–1.558)	0.428	0.75 (0.355–1.581)	0.449
Model 2: Trans ischemic attack					
	Smoking	1.109 (0.593–2.072)	0.746	1.133 (0.604–2.125)	0.696
	Hypertension	1.483 (0.698–3.15)	0.306	1.497 (0.703–3.187)	0.296

Model 1: F=1.553; Adjusted R2=0.006; Model 2: F=4.010; Adjusted R2=0.033; β_0_: unstandardized coefficient; β_1_: standardized coefficient; CI: confidence interval; OR: odds ratio; SE: standard error; VIF: variance inflation factor.

## DISCUSSION

This study evaluated the effect of CPO on the outcomes of CEA. It was noteworthy that CPO significantly reduced the need for intraoperative shunting and shortened both CC and total operative durations. Furthermore, timely detection of cerebral desaturation allowed prompt intraoperative interventions, which likely contributed to the reduction in neurological complications. These results support the growing body of evidence advocating for the integration of cerebral oximetry in carotid surgery to enhance patient safety and optimize surgical outcomes.

The first major finding of the study was the substantial decrease in shunt usage among patients monitored with CPO. Real-time monitoring of cerebral oxygen saturation provided critical data that guided intraoperative decision-making. The capacity of cerebral oximetry to detect cerebral desaturation acts as an early warning mechanism, enabling immediate interventions such as increasing blood pressure or administering neuroprotective agents. This observation aligns with previous studies highlighting the use of cerebral oximetry as an effective intraoperative monitoring technology in both cardiac and vascular surgeries^7^. The implementation of cerebral oximetry has the potential to significantly reduce the reliance on intraluminal shunting traditionally employed to prevent cerebral ischemia during carotid artery clamping^8^. A notable benefit of this approach is the associated decrease in shunt-related complications. By limiting the routine use of shunts, the incidence of adverse events such as air and particulate embolism is significantly reduced^9^. Although these complications are relatively uncommon, their potential for serious neurological sequelae underscores the importance of strategies to minimize their occurrence^10^. This paradigm aligns with contemporary surgical trends favoring less invasive interventions and associated risks^11^.

The study also demonstrated a reduction in carotid CC duration and total operative time. On average, CC time was decreased by approximately 10 min, contributing directly to shorter overall surgical duration. This reduction in operative time not only reduces risks associated with prolonged anesthesia exposure but also enhances procedural efficiency^12^. The reduction in operation time and anesthesia exposure contributes to improved cost-efficiency. Considering the neurological benefits of oximetry and the potential side effects associated with shunting, the use of oximetry appears to be a more cost-effective approach^13^.

In addition, the findings suggest that routine shunt placement may not be necessary in patients with significant unilateral carotid stenosis or occlusion undergoing contralateral CEA. Even in these high-risk cases, cerebral oximetry appeared to provide sufficient intraoperative guidance to safely forego shunting. This observation supports a more individualized approach to perioperative management, guided by real-time cerebral oxygenation monitoring^14^. It also aligns with the broader movement in surgical practice toward personalized, patient-specific strategies^15^.

The safety and efficacy of the CPO-guided approach are further underscored by the absence of mortality in this study cohort. A zero-mortality rate represents a significant outcome, reinforcing the potential of cerebral oximetry to enhance perioperative safety during CEA^16^. Moreover, these findings suggest that cerebral oximetry may contribute to broader improvements in patient outcomes across cardiovascular surgical procedures^17^. A systematic meta-analysis reported postoperative 30-day mortality and CVA rates after CEA. According to the results, the stroke rate was reported as 1.8% and the mortality rate as 0.6%. Our results were similar to this meta-analysis. No significant difference was found between the shunt usage and monitoring strategies compared in the studies examined in the meta-analysis^18^.

Various methods have been used to monitor ischemia during surgical and endovascular procedures, including neurocognitive assessment of the awake patient, measurement of reflux pressure in the carotid artery, somatosensory evoked potentials (SSEP), motor evoked potentials, electroencephalography (EEG), transcranial Doppler (TCD), and near-infrared spectroscopy. However, there is no consensus on which method is the gold standard or whether one is superior to the others^19-22^.

The limitation of the use of the CPO device used in our study can be mentioned as providing information only about the saturation in the frontal region blood flow. On the other hand, the fact that it allows for instant cerebral monitoring in a non-invasive manner provides a great advantage in terms of surgical procedure complications. In the study of Jahangiri et al., where the combined monitoring with EEG, TCD, and SSEP was presented, it was stated that possible brain ischemia was prevented with the joint use of all these tools. However, in these studies, it was stated that the placement and evaluation of both EEG and TCD probes required an expert, that they caused the surgical intervention to be delayed, and that distal probes should be connected for SSEP, and ischemia control should be done with stimuli. In addition, the anesthesia and surgical team should be warned during and after each procedure. The fact that all these procedures depend on many components brings their usability into question. In our study, the surgical procedure can be completed with morbidity and mortality compatible with the literature by using only CPO, and the postoperative 30-day follow-ups are considered compatible with the literature^23^.

In conclusion, integrating CPO into CEA represents a promising strategy for enhancing patient safety, decreasing operative duration, and potentially improving overall clinical outcomes. Its ability to guide intraoperative decision-making—especially about selective shunt use—marks a significant advancement in vascular surgery. Nonetheless, the current study's limitations underscore the need for further research to validate these findings and assess their broader impact within routine clinical practice.

Nonetheless, several limitations must be acknowledged. The observational study design and relatively small sample size restrict the generalizability of the findings. The study also focused on short-term outcomes, with no data on the long-term effects of a cerebral oximetry-guided approach in CEA. Further investigation is required to assess the durability of these outcomes over time. In addition, the separation of patients into groups within the scope of this study necessarily consists of two different periods. To address these limitations, future research should include larger, more diverse patient populations and ideally employ randomized controlled trial designs. Such studies are essential to generate more robust evidence, enabling stronger causal inferences and a clearer understanding of the applicability of these findings across diverse clinical settings. Additionally, future research should evaluate the cost-effectiveness of CPO in CEA, particularly in light of its potential to reduce operative time and perioperative complications.

## CONCLUSION

The clinical value of CPO is demonstrated in this study, especially in terms of lowering the frequency of intraoperative shunting. This non-invasive technique makes it possible to identify cerebral desaturation in real time, which enables surgeons to administer hemodynamic or pharmacological treatments in a timely manner to maintain sufficient cerebral perfusion without the need for a shunt. By lowering the frequency of shunt-related issues, including embolism and vascular damage, this method improves patient safety. Furthermore, lower CC and total operating times—two clinically significant markers of surgical effectiveness and decreased patient risk—were linked to the use of cerebral oximetry. Cerebral oximetry successfully guided surgical decisions in patients with unilateral carotid stenosis or occlusion undergoing contralateral CEA, preventing needless shunt installation. Our cohort's low complication rate and lack of perioperative mortality provide credence to the method's clinical safety and viability.

All of our results point to the possibility that regular incorporation of CPO into CEA treatments could result in safer, more customized surgical approaches, which would ultimately enhance immediate results and maximize the use of resources in vascular surgery departments.

## Data Availability

The datasets generated and/or analyzed during the current study are available from the corresponding author upon reasonable request.
